# Identification of microRNA-mRNA functional interactions in UVB-induced senescence of human diploid fibroblasts

**DOI:** 10.1186/1471-2164-14-224

**Published:** 2013-04-04

**Authors:** Ruth Greussing, Matthias Hackl, Pornpimol Charoentong, Alexander Pauck, Rossella Monteforte, Maria Cavinato, Edith Hofer, Marcel Scheideler, Michael Neuhaus, Lucia Micutkova, Christoph Mueck, Zlatko Trajanoski, Johannes Grillari, Pidder Jansen-Dürr

**Affiliations:** 1 Institute for Biomedical Aging Research, Austrian Academy of Sciences, Rennweg 10, Innsbruck 6020, Austria; 2 Department of Biotechnology, BOKU - University of Natural Resources and Life Sciences Vienna, Muthgasse 18, Vienna 1190, Austria; 3 Division for Bioinformatics, Biocenter, Medical University Innsbruck, Innrain 52, Innsbruck 6020, Austria; 4 Institute for Genomics and Bioinformatics, University of Technology, Petersgasse 14, Graz 8010, Austria; 5 Evercyte GmbH, Muthgasse 18, Vienna, A 1190, Austria

**Keywords:** Aging, Stress-induced senescence, miR-101, Ezh2, Epigenetics, UVB

## Abstract

**Background:**

Cellular senescence can be induced by a variety of extrinsic stimuli, and sustained exposure to sunlight is a key factor in photoaging of the skin. Accordingly, irradiation of skin fibroblasts by UVB light triggers cellular senescence, which is thought to contribute to extrinsic skin aging, although molecular mechanisms are incompletely understood. Here, we addressed molecular mechanisms underlying UVB induced senescence of human diploid fibroblasts.

**Results:**

We observed a parallel activation of the p53/p21^WAF1^ and p16^INK4a^/pRb pathways. Using genome-wide transcriptome analysis, we identified a transcriptional signature of UVB-induced senescence that was conserved in three independent strains of human diploid fibroblasts (HDF) from skin. In parallel, a comprehensive screen for microRNAs regulated during UVB-induced senescence was performed which identified five microRNAs that are significantly regulated during the process. Bioinformatic analysis of miRNA-mRNA networks was performed to identify new functional mRNA targets with high confidence for miR-15a, miR-20a, miR-20b, miR-93, and miR-101. Already known targets of these miRNAs were identified in each case, validating the approach. Several new targets were identified for all of these miRNAs, with the potential to provide new insight in the process of UVB-induced senescence at a genome-wide level. Subsequent analysis was focused on miR-101 and its putative target gene Ezh2. We confirmed that Ezh2 is regulated by miR-101 in human fibroblasts, and found that both overexpression of miR-101 and downregulation of Ezh2 independently induce senescence in the absence of UVB irradiation. However, the downregulation of miR-101 was not sufficient to block the phenotype of UVB-induced senescence, suggesting that other UVB-induced processes induce the senescence response in a pathway redundant with upregulation of miR-101.

**Conclusion:**

We performed a comprehensive screen for UVB-regulated microRNAs in human diploid fibroblasts, and identified a network of miRNA-mRNA interactions mediating UVB-induced senescence. In addition, miR-101 and Ezh2 were identified as key players in UVB-induced senescence of HDF.

## Background

Cellular senescence is controlled through a variety of regulatory mechanisms with particular contribution of the p53 [1] and Rb [2] tumor suppressor pathways. Briefly, p53 is activated in response to telomere damage or other kinds of DNA damage and orchestrates the transcriptional activation of a variety of downstream genes contributing to growth arrest, such as the CDK inhibitor p21^WAF1^. In a parallel pathway, upregulation of the CDK inhibitor p16^INK4a^ leads to the inhibition of phosphorylation of the retinoblastoma protein (pRb), thereby enforcing prolonged cell cycle arrest [3], which is also a useful marker for senescence in human tissues [4]. Activation of p16^INK4a^ triggers the appearance of senescence-associated heterochromatin foci [5], at least in some cell types. Activation of the p53/p21^WAF1^ and p16^INK4a^/pRb pathways represent two alternative scenarios for senescence initiation, and there is evidence that parallel activation of both pathways leads to enforced senescence [6], also referred to as “deep senescence” [7]. The relative importance of each of these pathways for cellular senescence seems to depend on the cell type. It should be noted that, besides these central pathways, a variety of signals have been shown to initiate a senescence response [8].

Stress-induced premature senescence (SIPS) has been identified as a model for telomere-independent senescence that can be initiated by various forms of stress, including oxidative stress, irradiation, replicative stress and oncogene activation [9]. In the case of skin derived human diploid fibroblasts (HDF), repeated mild treatment with UVB has been established as an experimental model for extrinsic skin aging [10], which depends on the accumulation of senescent cells, in particular fibroblasts in the dermis [9]. UVB irradiation is known to induce aspects of a DNA damage response, and it was reported that knocking down p53 alleviates but does not abrogate the senescence response of HDF to repeated UVB stress [11], suggesting that both p53-dependent and -independent pathways may cooperate to enforce UVB-induced senescence. However, UVB irradiation is known to affect a variety of intracellular signal transduction pathways [12,13] and the precise sequence of events during establishment of UVB-induced premature senescence remained elusive.

Recent studies have emphasized an important role of non-coding RNAs, also referred to as microRNAs, as regulators of gene expression [14]. MicroRNAs are generated from larger precursor RNAs and were shown to interfere with the expression of protein coding genes by several mechanisms, including i) destabilization of specific mRNAs and ii) prevention of translation of specific mRNAs. Messenger RNAs targeted by microRNAs usually contain short sequences of homology [15]. It is known that changes in microRNA expression are contributing to cellular senescence [16] and organismic aging [17,18]; however, the role of microRNAs, if any, in UVB-induced senescence of human fibroblasts is only poorly understood. In the present communication, we have addressed molecular mechanisms underlying the establishment of premature senescence in human fibroblasts exposed to repeated series of mild UVB irradiations, as a model system to monitor molecular processes involved in extrinsic skin aging. We identified a small set of microRNAs, which are differentially regulated during the process and performed transcriptome analysis, in combination with advanced bioinformatics, to identify potential targets for these microRNAs.

## Results

### UVB-induced changes in gene expression

Human diploid fibroblasts (HDF, strain HFF-2) derived from newborn foreskin were subjected to eight consecutive UVB treatments of 4000 J/m^2^ during four days. Under these conditions no overt cell death was observed (data not shown). Cell proliferation was strongly inhibited, and UVB-treated cells performed less than 2 population doublings (PDL) over 15 days of the experiment, whereas untreated cells underwent 12 PDL in the same time period. The growth arrest phenotype reached by repeated mild UVB stress resembled cellular senescence, as judged by the changes of cell morphology and the accumulation of a large percentage of cells that stained positive for senescence-associated β-galactosidase (SA-ß-gal) (Additional file 1: Figure S1). As could be expected, UVB treatment led to a strong but transient phosphorylation of p53 on serine 15, most notable at day 4, i.e. after application of the last stress, indicative of p53 activation, consistent with previous observations [19]. The overall levels of p53 protein were also increased, in line with previous findings [20]. Activation of p53 also resulted in the upregulation of its downstream effector p21^WAF1^, which is known to enforce cellular senescence in response to DNA damage [21]. We also addressed effects of UVB treatment on the p16^INK4A^/pRb pathway, representing the other important pathway relevant for cellular senescence [22]. As could be expected from the observed upregulation of p21^WAF1^, UVB treatment led to the rapid disappearance of phosphorylated species of the retinoblastoma protein (pRb), which was accompanied by a delayed but significant upregulation of the CDK inhibitor p16^INK4A^ (Additional file 1: Figure S1). Together the data suggest that mild UVB stress induces premature cellular senescence and that both the p53/p21^WAF1^ axis and the p16^INK4A^/pRb pathway are involved in the senescence response, consistent with previous reports [23-25]. The same protocol of repeated mild UVB stress induced premature senescence also in two other strains of human diploid fibroblasts, HFF-1 and PFF, with a very similar kinetics (Additional file 2: Figure S2), along with the activation of the p53/p21^WAF1^ and pRb/p16^INK4A^ pathways (Additional file 3: Figure S3).

**Figure 1:**
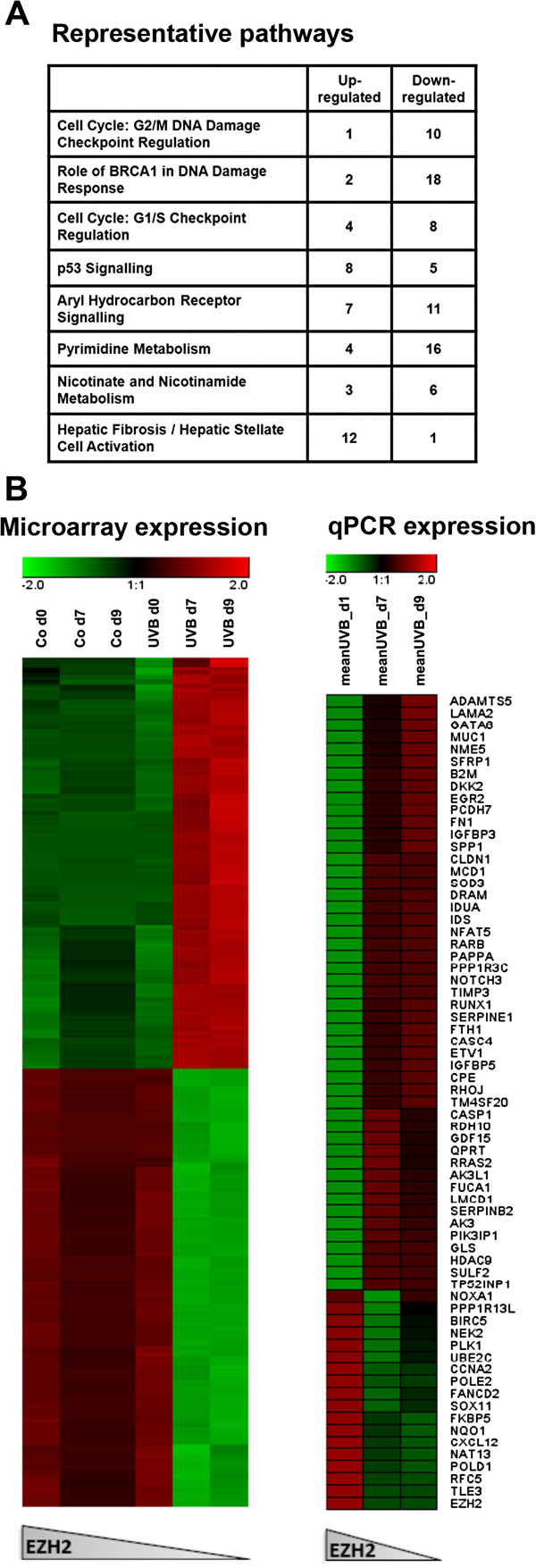
**Transcriptome analysis of UVB-induced senescence. A**. Ingenuity pathway analysis. Activated pathways were determined by Ingenuity System Pathway Analysis software (http://www.ingenuity.com/) by Core Analysis. Shown are eight significantly activated pathways (threshold p-value ≤ 0.05). **B**. *In silico* analysis of mRNA expression change in response to UVB irradiation. *(left)* The differential gene expression profile from total RNA was examined by Affymetrix GeneChip analysis (cutoff of > 1.5 fold regulation) 1, 7, and 9 days between control and UVB irradiated of HDF samples. The expression values were sorted by level of Ezh2 expression (rows). *(right)* Heatmap of selected 67 genes were determined by RT-PCR and sorted by level of Ezh2 expression (rows), red representing overexpression and green representing underexpression of the transcript.

To characterize the response of HDF to UVB-induced premature senescence, genome-wide transcriptome analysis was performed at various time points. Applying a cutoff of > 1.5 fold regulation, this analysis revealed the upregulation of 1219 genes and the downregulation of another 1077 genes in response to mild UVB treatment. Changes in gene expression level were most pronounced at days 7 and 9 after beginning of the treatment. Initial analysis of UVB-responsive pathways by Ingenuity™ Software, based on the results of Affymetrix chip analyses, revealed several distinct molecular pathways preferentially affected by the treatment, including G1/S cell cycle checkpoint, DNA damage checkpoint, p53 signaling pathway, cell migration, aryl hydrocarbon receptor signaling, pyrimidine metabolism and nicotinate and nicotinamide metabolism (Figure 1A). For day 9 after UVB irradiation, three independent Affymetrix chip experiments were performed, which revealed significant upregulation (p < 0.05) for a total of 632 genes, whereas 716 genes were significantly downregulated (p < 0.05). The list of all regulated genes and corresponding Affymetrix expression data are provided as Additional file 4: Table S1. From these genes, a set of 67 genes was selected for an independent analysis by RT-qPCR, which confirmed all regulations revealed by Affymetrix chips (Figure 1B).

### microRNAs regulated in UVB-induced senescence

**Figure 2:**
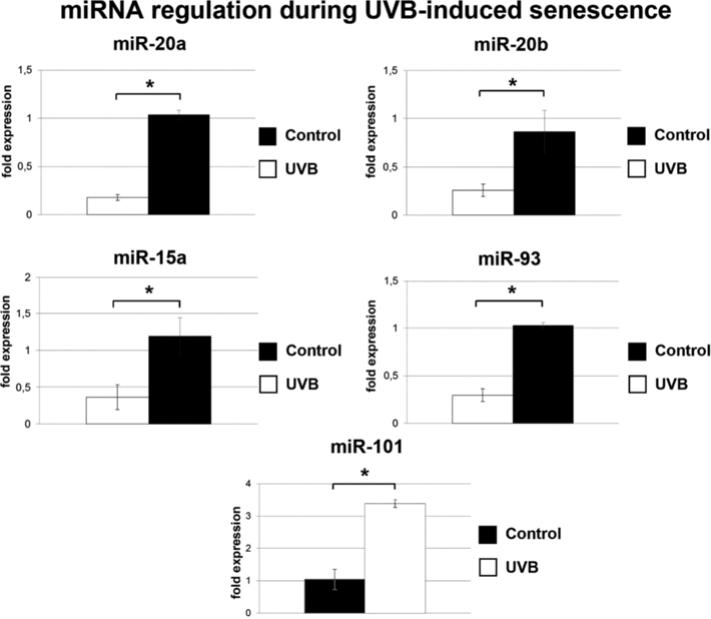
**Validated miRNA expression levels.** miRNA was isolated from UVB treated and control cells. miRNA expression levels for miR-20a, miR-20b, miR-15a, and miR-93 were determined by Locked nucleic acid (LNA)-miRNA microarray. Experiments were performed in triplicates. *p < 0.01; **p < 0.001.

**Figure 3:**
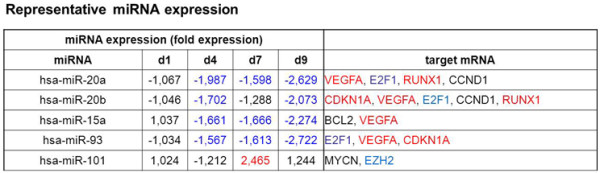
**microRNA expression level.** The expression levels of 806 miRNAs were determined at day 1, 4, 7 and 9 by LNA microarray. Displayed are five selected miRNAs with the corresponding mRNA targets, identified by DIANA LAB (http://diana.cslab.ece.ntua.gr/tarbase/) and miRecords (http://mirecords.biolead.org/). In red are miRNAs and target mRNAs that are upregulated, downregulated genes are colored in blue and not regulated genes are in black.

We also determined the expression levels of 806 miRNAs in UVB treated compared to control cells. The relative expression levels of miRNAs at various time points after UVB stress, as determined by miRNA array, are shown in Additional file 5: Table S2. We used publicly available and in-house developed tools, to compare expression profiles of all miRNAs and the 67 validated mRNAs (Figure 1B), and predicted target pairs to score and identify high confidence miRNA targets. Thereby, eight miRNAs (miR-15a, miR-17, miR-20a, miR-20b, miR-34, miR-93, miR-101, miR-155) were identified for which regulated mRNA targets were found with high confidence. Subsequently, the expression levels of selected miRNAs were analyzed by qPCR. In these experiments, data obtained by the miRNA array for miR-15a, miR-20a, miR-20b, miR-93, and miR-101 were confirmed (Figure 2); whereas miR-17, miR-34 and miR-155 were also regulated in UVB-treated cells in accordance with the miRNA array results, the observed differences did not reach statistical significance (data not shown). Expression levels for miR-15a, miR-20a, miR-20b, miR-93, and miR-101 are shown in Figure 3, along with their established target mRNAs.

### miRNA-mRNA regulatory networks in UVB-induced senescence

**Figure 4:**
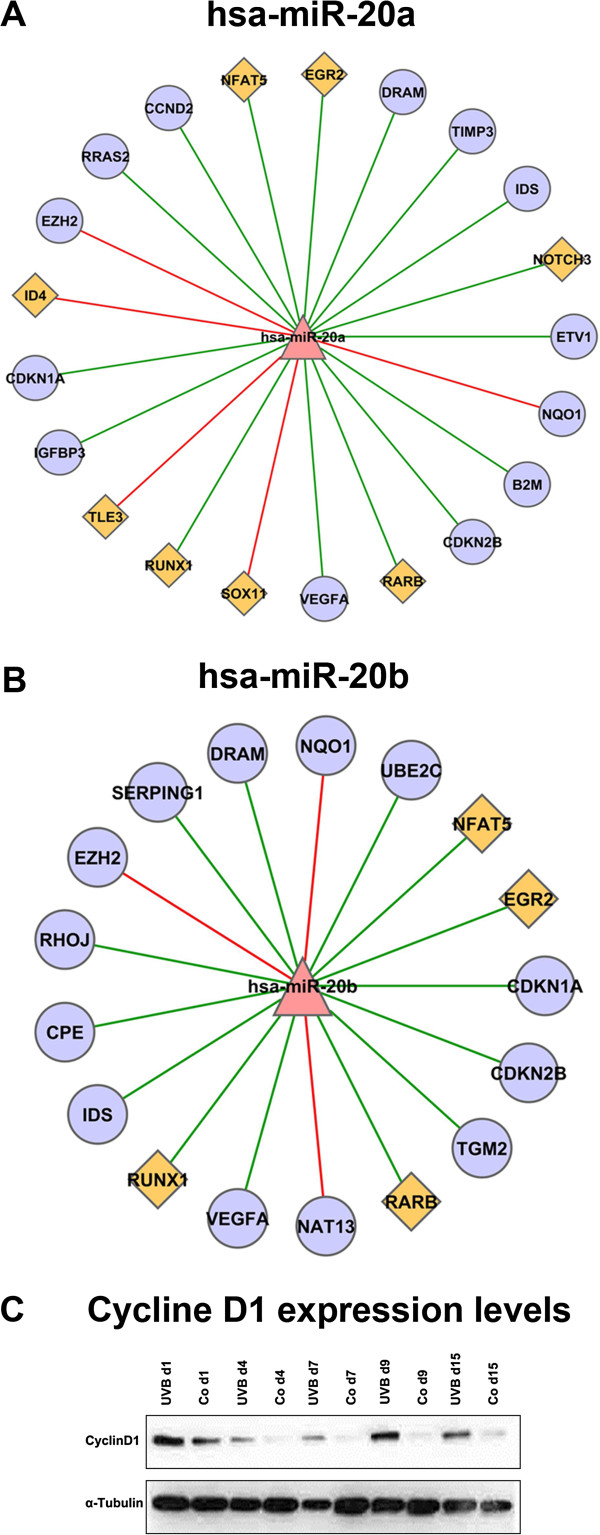
**Correlation network of miR-20a/b and their high confidence target genes.** We used 10 prediction tools to obtain, based on public data, candidate miRNA-mRNA target interactions and we identified high confidence targets by mRNA and miRNA expression. In positive cases, miRNA expression should show a negative correlation with the respective target gene mRNA level. We calculated Pearson correlation coefficients between miRNAs and their targets. Results of the analysis are presented here for miR-20a **(A)**, and miR-20b **(B)**. The color and shape of nodes are based on different node attributes available for the analyzed dataset. The red triangles, purple circles and orange diamonds in the network are indicating miRNAs, target genes, and transcription factors, respectively. Edges represent correlation between miRNAs and mRNAs, the color of the edges designate the type of interaction. Red is for positive and green is for negative correlation. Protein was isolated from UVB irradiated and control cells at the indicated time points. Protein levels were analyzed by standard Western blot for Cyclin D1 **(C)**. Experiments were performed in triplicates, one representative experiment is shown.

High confidence targets were identified for miR-20a and miR-20b encoded by the miR-17-92 cluster which is known to synergize with Myc in cancer development [26]*,* probably through repression of p21^WAF1^ expression at the post-transcriptional level [27]. However, there is also evidence that miR-17-92 blocks E2F-dependent steps in the regulation of angiogenesis [28]. Our analysis confirmed a high confidence interaction between both miR-20a and miR-20b with p21^WAF1^ (CDKN1A), p15^INK4B^ (CDKN2B), RUNX1, and vEGF-A (Figure 4A,B), thereby validating the analytical procedure. E2F1 and Cyclin D1 are predicted targets for both miR-20a and miR-20b. Whereas decreased expression of miR-20a/b was not correlated with altered mRNA levels of E2F1 and Cyclin D1 (Figure 4A,B), upregulation of Cyclin D1 gene expression during UVB-induced senescence was observed at the protein level (Figure 4C). Results of the bioinformatic analysis suggested several so far unreported potential targets for miR-20a and miR-20b in the context of UVB-induced senescence. Thus, DRAM, IDS, NFAT5, EGR2, CCND2, and RARB were identified as potential high confidence interactions for both miR-20a and miR-20b (Figure 4A,B). In addition, the data suggest TIMP3, ETV1, B2M, IGFBP-3, and RRAS2 as potential targets for miR-20a (Figure 4A), and TGM2, CPE, RHOJ, and SERPING1 as potential targets for miR-20b (Figure 4B).

**Figure 5:**
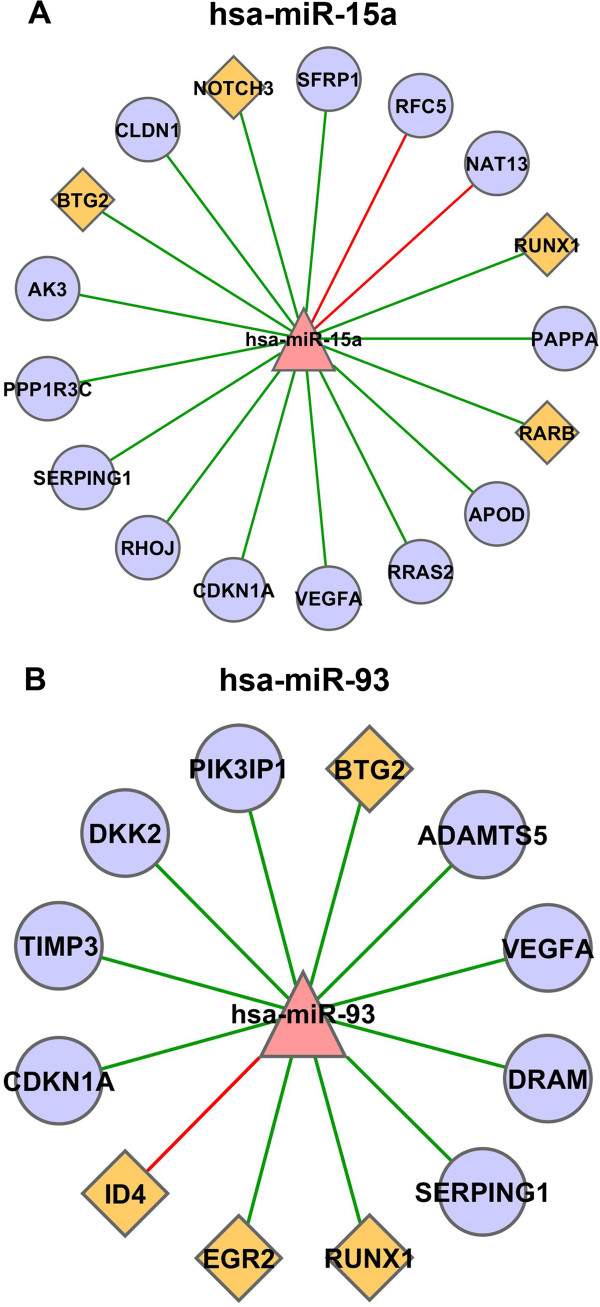
**Correlation networks of miR-93/miR-15 and their high confidence target genes.** We used 10 prediction tools to obtain, based on public data, candidate miRNA-mRNA target interactions and we identified high confidence targets by mRNA and miRNA expression. We calculated Pearson correlation coefficients between miRNAs and their targets. Results of the analysis are presented here for miR-93 **(A)**, and miR-15 **(B)**. The color and shape of nodes are based on different node attributes available for the analyzed dataset. The red triangles, purple circles and orange diamonds in the network are indicating miRNAs, target genes, and transcription factors, respectively. Edges represent correlation between miRNAs and mRNAs, the color of the edges designate the type of interaction. Red is for positive and green is for negative correlation.

miR-93 is known to inhibit angiogenesis by suppressing VEGF release [29], and contributes to silencing of p21^WAF1^ gene expression after DNA damage [30]. Moreover, miR-93 increases survival in cisplatin-resistant ovarian cancer cells, by directly targeting PTEN and upregulation of the AKT signaling pathway [31]. Our analysis in UVB-induced senescence confirmed a high confidence interaction of miR-93 with CDKN1A and vEGF-A (Figure 5A), thereby validating the analytical procedure. Results of the bioinformatic analysis suggested DRAM, PIK3IP1, DKK2, Serpin G1, ADAMTS5, TIMP3, BTG2, RUNX1 and EGR2 as potential targets for miR-93 (Figure 5A).

miR-15a, along with miR-16, is commonly deleted in human chronic lymphocytic leukemia [32] and known to target multiple oncogenes, including BCL2, MCL1, CCND1, and WNT3A [33]. Our analysis confirmed a high confidence interaction between miR-15a and vEGF-A in UVB-induced senescence (Figure 5B), thereby validating the analytical procedure. Results of the bioinformatic analysis suggested PAPPA, APOD, RRAS2, Runx1, RARB, BTG2, Notch3 and SFRP1 as potential targets for miR-15 (Figure 5B).

**Figure 6:**
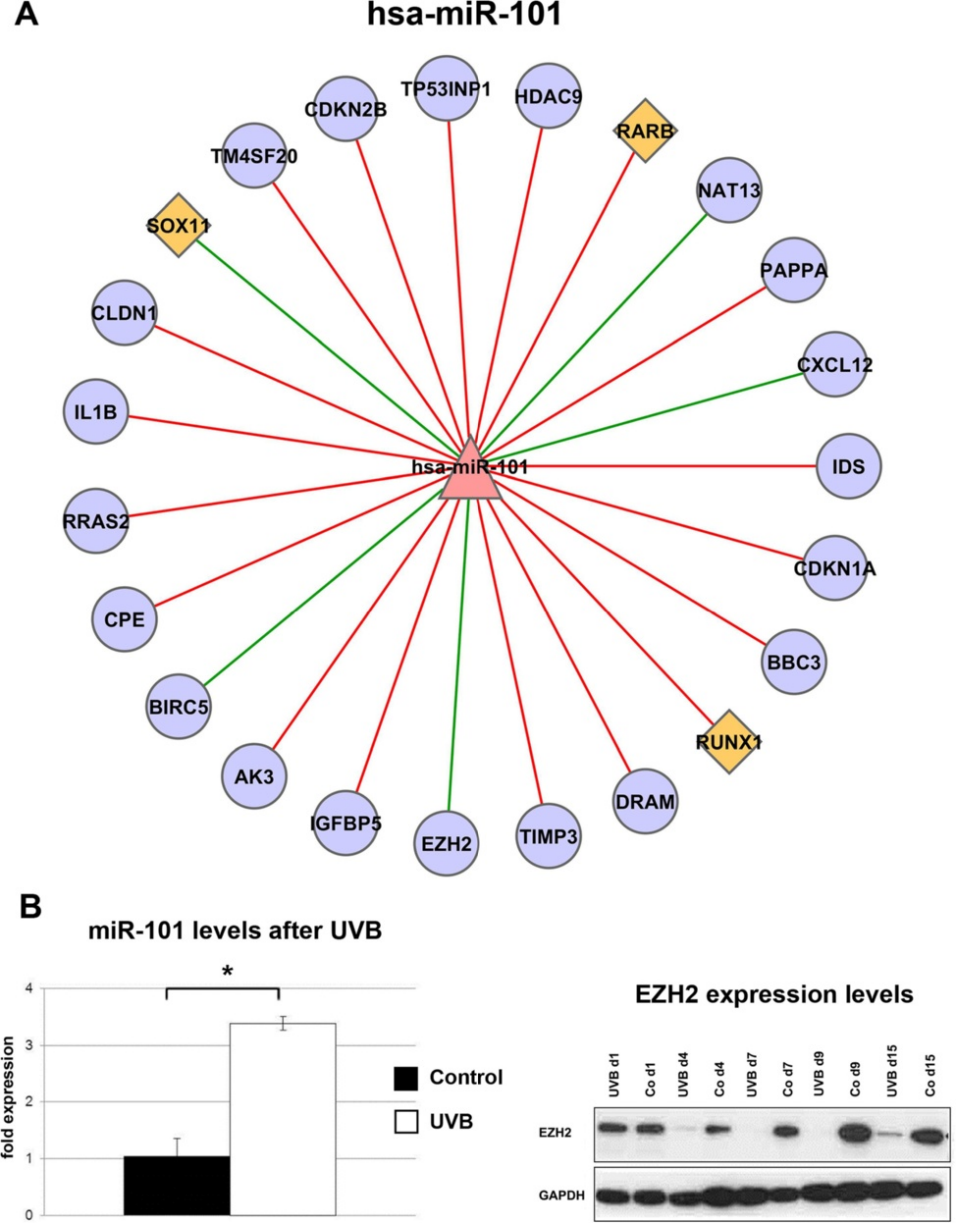
**Correlation network of miR-101 and its high confidence target genes. (A)** We used 10 prediction tools to obtain, based on public data, candidate miRNA-mRNA target interactions and we identified high confidence targets by mRNA and miRNA expression. We calculated Pearson correlation coefficients between miRNAs and their targets. Results of the analysis are presented here for miR-101. The color and shape of nodes are based on different node attributes available for the analyzed dataset. The red triangles, purple circles and orange diamonds in the network are indicating miRNAs, target genes, and transcription factors, respectively. Edges represent correlation between miRNAs and mRNAs, the color of the edges designate the type of interaction. Red is for positive and green is for negative correlation. **(B)** Protein samples for the indicated time points were collected as described (left panel). Protein levels were determined for Ezh2 by standard Western blot analysis. Experiments were performed in triplicates, shown here is a representative result. miR-101 levels were determined by real-time qPCR as described (right panel). Data represents the mean ± SE for three independent experiments. Co: untreated controls. *p < 0.01; **p < 0.001.

miR-101 is known to suppress expression of the histone methyltransferase Ezh2 [34,35]. Our analysis confirmed a high confidence interaction between miR-101 and Ezh2 in UVB-induced senescence (Figure 6A), thereby validating the analytical procedure. Accordingly, Ezh2 expression was significantly downregulated at both mRNA and protein level in UVB treated cells (Figure 6B). Results of the bioinformatic analysis suggested BIRC5, NAT13, and CXCL12 as potential targets for miR-101. (Figure 6A). Whereas the majority of analyzed potential mRNA targets for miR-101 displayed a positive correlation with miR-101 levels (red lines in Figure 6A), the biological meaning of these interactions remains to be established.

To further validate the bioinformatics-based target selection, regulation of several newly identified candidate genes by specific microRNAs was addressed in HDF overexpressing miR-15a, miR-20a, and miR-93, respectively. In all cases, microRNA levels were significantly ( > 20 fold) increased by transfection (data not shown). For the genes RARB, RUNX1 and CDKN2B, we found that overexpression of the appropriate microRNA species (miR-15a and miR-20a, respectively) reduced protein levels of the respective gene products (Additional file 6: Figure S4). Whereas these findings do not prove binding of microRNAs 15a and 20a to the 3'-UTR of the target genes, downregulation of protein expression by overexpression of selected miRNAs provides a functional validation of the bioinformatics approach. The mRNAs for several other genes, including VEGFA, RHOJ, and NOTCH3 were significantly down-regulated by the appropriate microRNAs (data not shown); however, the limited availability of high-quality antibodies precluded determination of protein expression levels in these cases.

### A role of miR-101/Ezh2 in UVB-induced senescence?

**Figure 7:**
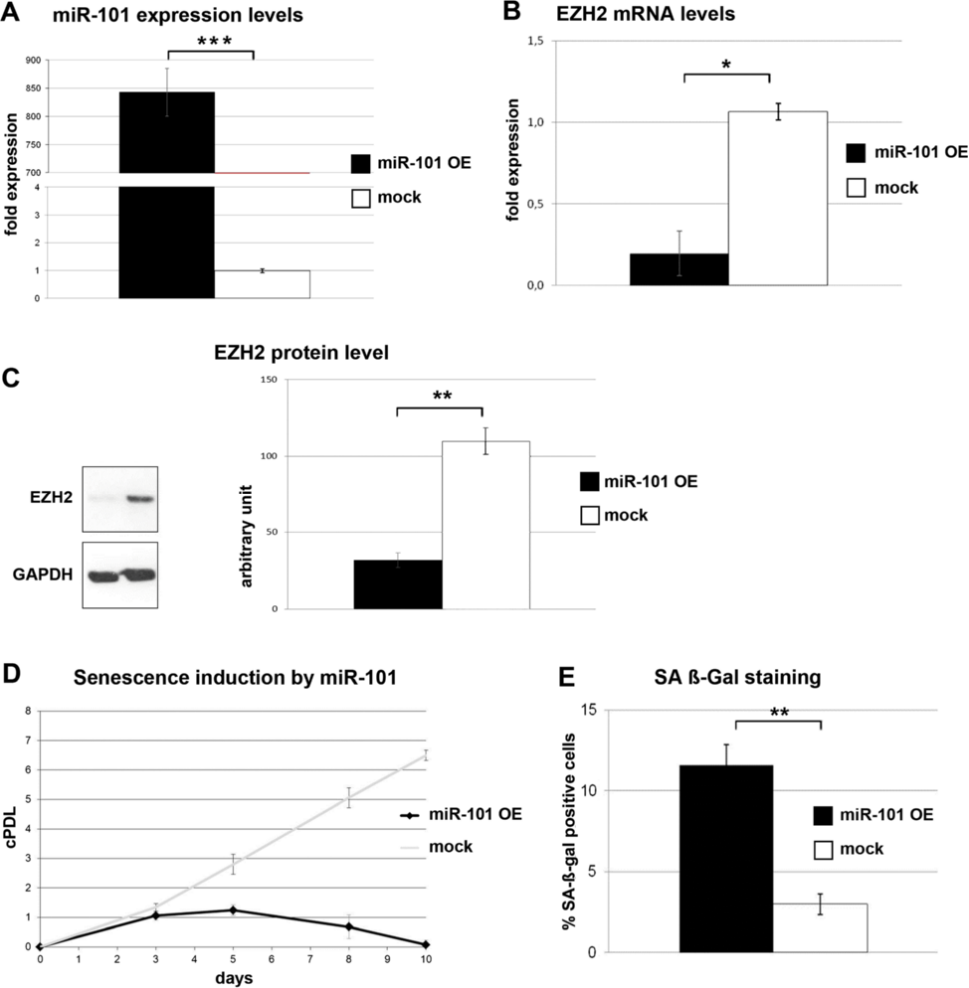
**miR-101 overexpression induces premature senescence in HDFs.** Cells were reverse transfected as described with miR-101 precursors for overexpression, negative control or with the transfection reagent (siPORT™ *NeoFX™*) at day 0, 3, 5 and 7. At day 9 RNA and protein were isolated. Expression levels of **(A)** miR-101 and **(B)** Ezh2 mRNA were determined by real-time qPCR. Data represents the mean ± SE for three independent experiments. **(C)** Standard Western blot analysis was performed with monoclonal mouse anti-Ezh2 antibody. Left panel represents densitometric data calculated out of three independent experiments (± SD). **(D)** Growth curve analysis of miR-101 overexpressing HDFs. cPDLs were calculated. Data represents the mean ± SD for three independent experiments. **(E)** To determine the senescence status of miR-101 overexpressing and control HDFs, cells were stained for SA-β-gal at day 9. Bars represent the relative amount of SA-β-gal positive cells (± SD). OE: overexpression; cPDL: cumulative population doublings. *p < 0.01; **p < 0.001.

To address the functionality of the miR-101-Ezh2 interaction in UVB-induced senescence of HDF and their importance for UVB-induced senescence, we addressed the potential of these molecules to affect cellular senescence in HDF. In a first set of experiments, we analyzed consequences of overexpression of miR-101. Using reverse transfection, miR-101 was overexpressed in human diploid fibroblasts, which resulted in a clear upregulation of miR-101 (Figure 7A). The strong upregulation of miR-101 induced a significant downregulation in the level of Ezh2 mRNA (Figure 7B) and protein (Figure 7C). In consequence, overexpression of miR-101 was sufficient to reduce the rate of proliferation of human diploid fibroblasts (Figure 7D) and induced a significant increase in the number of SA-β-gal positive cells (Figure 7E). These experiments suggest that indeed miR-101 has the potential to downregulate Ezh2 mRNA and protein levels in HDF, and this can lead to growth arrest and entry into premature senescence. To knock down Ezh2 expression in HDF, lentiviral vectors carrying Ezh2-targeting shRNAs were used. Out of five shRNAs tested, #73 and #75 effected a significant downregulation of Ezh2 levels in transfected cells (Additional file 7: Figure S5). Lentiviral vectors were prepared carrying these shRNAs and used to infect HDF. Ezh2 knockdown led to a downregulation of cell proliferation, along with a significant increase in the percentage of SA-β-gal positive cells, suggesting that knocking down Ezh2 can induce premature senescence of HDF (Additional file 7: Figure S5).

**Figure 8:**
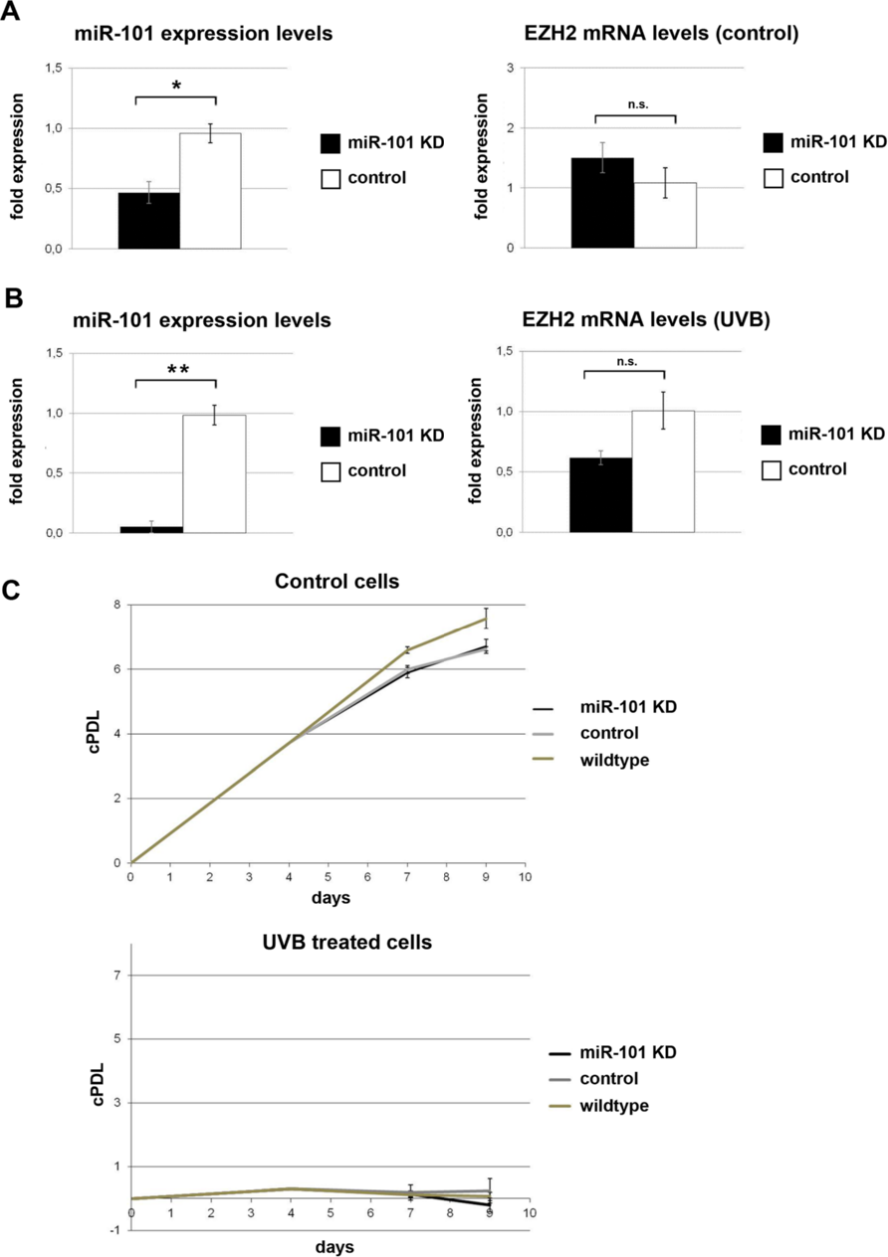
**Depletion of miR-101 fails to prevent UVB-induced senescence.** Cells were irradiated with UVB twice a day for 4 days. After the last exposure the cells were reverse transfected as described with miR-101 power inhibitors for knockdown, negative control or not treated. Reverse transfection was repeated at day 7. At day 9 RNA and protein were isolated. **A**. Control cells. Expression levels of miR-101 and Ezh2 mRNA of non-irradiated cells after miR-101 knockdown were determined by real-time qPCR. Bars indicate the mean ± SE of three independent experiments. **B**. UVB-treated cells miR-101 and Ezh2 expression levels from UVB treated cells after miR-101 knockdown were determined by real-time qPCR. Bars indicate the mean ± SE of three independent experiments. **C**. Growth curve analysis of miR-101 knockdown cells. cPDLs were calculated as described. Data represent the mean ± SD of three independent experiments. cPDL: cumulative population doublings KD: knockdown. *p < 0.01; **p < 0.001.

To address the role of miR-101 in UVB-induced cellular senescence, we attempted to experimentally reduce miR-101 levels by transfection of miR-101 inhibitory RNAs. This treatment was started one day before UVB treatment and continued thereafter, in order to keep miR-101 levels constantly low. Using reverse transfection, the levels of miR-101 were significantly reduced in control HDF (Figure 8A) and miR-101 knockdown efficiently abrogated miR-101 upregulation in UVB-treated fibroblasts (Figure 8B). However, Ezh2 mRNA was not significantly upregulated in miR-101 depleted cells, irrespective of UVB treatment (Figure 8A,B); and miR-101 knockdown failed to increase Ezh2 protein levels in both cases (data not shown). To address potential mechanisms underlying the failure to upregulate Ezh2, Ezh2 was also overexpressed by lentiviral vectors. Both in HDF and in easy-to-transfect human osteosarcoma (U2-OS) cells, overexpression of Ezh2 mRNA was transient and no elevation of Ezh2 protein levels was observed in HDF (Additional file 8: Figure S6), for reasons that remain to be established. Together, these experiments indicate that reduction of miR-101 was not sufficient to rescue Ezh2 expression in UVB-treated cells. Accordingly, the phenotype of UVB-induced cellular senescence was not significantly affected by preventing the UVB-induced upregulation of miR-101 (Figure 8C). Consistent with this observation, knockdown of miR-101 also failed to significantly reduce the percentage of SA-β-gal positive cells after UVB treatment (data not shown).

## Discussion

In this communication, we addressed molecular mechanisms underlying UVB-induced senescence of human diploid fibroblasts. Using genome-wide transcriptome analysis, we identified a transcriptional signature of UVB-induced senescence. In parallel, a comprehensive screen for microRNAs regulated during UVB-induced senescence was performed and five microRNAs were identified that are significantly regulated during the process. Subsequent analysis revealed several well established miRNA-mRNA regulatory interactions including miR-101/Ezh2, thereby validating the assay. In addition, several new miRNA-mRNA regulatory interactions were identified to occur in UVB-induced senescence. Overexpression of miR-101 and downregulation of Ezh2 independently induced senescence in the absence of UVB irradiation. However, the downregulation of miR-101 was not sufficient to block the phenotype of UVB-induced senescence, suggesting that other UVB-induced processes contribute to the senescence response, partially redundant with upregulation of miR-101. These findings extend our knowledge of miR-mRNA regulatory interactions, and at the same time provide a rich resource for data mining to identify new players in UVB-induced senescence and potential targets for interventions in extrinsic skin aging.

### Transcriptional profiling of UVB-induced premature senescence

Whereas a low-density DNA array was used previously to study changes in the level of 240 senescence-related genes in UVB-induced senescence of HDF [11], the current study provides the first genome-wide transcriptional analysis of UVB-induced senescence. As could be expected [1], activation of p53 signaling and suppression of the G1/S transition were observed along with a pronounced DNA damage response, as revealed by pathway analysis tools. We also observed concerted regulation of genes in the aryl hydrocarbon receptor signaling and hepatic fibrosis/heaptic stellate activation pathways, which conceivably reflect specific responses to UVB irradiation, used here to trigger the senescence response. Of note, there were also distinct changes in the regulation of genes coding for enzymes in the pyrimidine and nicotinamide metabolism; however, more work will be required to fully understand the implications of these alterations for UVB-induced senescence. Conceivably, the transcriptional signature of UVB-induced senescence, as shown here, will pinpoint new targets for intervention in extrinsic photoaging, which will be investigated in follow-up studies.

### miRNA-mRNA regulatory networks in UVB-induced senescence

miR-34, a known transcriptional target of p53 [36], was strongly upregulated at day 7, as expected from the observed activation of p53. Similarly, downregulation of miR-20a and miR-20b is consistent with previous observations suggesting that members of the miR-17-92 cluster are commonly downregulated in various senescence models as well as in organismal aging [16]. These observations validated the miRNA screening approach. It is known that miRNAs regulate both stability and translation of mRNAs, and in most cases upregulation of miRNAs leads to the inhibition of gene function [37]. The miR-17-92 cluster, containing the microRNA-17-18-19-20-92 polycistron, cooperates with Myc in tumorigenesis [26], probably by silencing of p21^WAF1^ expression at the post-transcriptional level [27]. On the other hand, miR-17-92 inhibited proliferation and metastasis of pancreatic carcinoma cells [38], blocked E2F-dependent steps in the regulation of angiogenesis [28], and repressed endothelial cell migration [39], suggesting cell type specific effects. Our finding that E2F1 and VEGFA are relevant targets for miR-20a/b in the context of UVB-induced senescence is consistent with these data.

Although frequently overexpressed in human malignancies, miR-93 may actually function as a tumor suppressor gene. Accordingly, miR-93 abrogated VEGF protein secretion, suggesting that miR-93 interferes with angiogenesis [29], blocked tumor development in mammary fat pads [40], and suppressed proliferation of human colon cancer stem cells [41]. However, miR-93 was also shown to i) promote tumor growth and angiogenesis by targeting integrin-β8 [42], ii) to contribute to silencing of p21^WAF1^ gene expression after DNA damage [30], and iii) to promote cell proliferation and clonogenicity of HepG2 Cells [43], suggesting that effects of miR-93 depend on cellular context. Upregulation of p21^WAF1^ gene expression in UVB-treated cells was correlated with the downregulation of miRNAs 20a/b and 93, known to target p21^WAF1^[27,30,44-46], and the identification of E2F1 and VEGFA as relevant targets for miR-93 in the context of UVB-induced senescence is consistent with the available data.

miR-15a, along with miR-16, was the first microRNA linked to cancer because both genes are commonly deleted in human chronic lymphocytic leukemia [32]. Expression of miRNAs encoded by the miR-15/16 cluster inhibits cell proliferation, promotes apoptosis of cancer cells, and suppresses tumorigenicity both *in vitro* and *in vivo*. miR-15a and miR-16-1 function by targeting multiple oncogenes, including BCL2, MCL1, CCND1, and WNT3A [33,47]. In contrast to their function as tumor suppressors, miR-15 can also promote tumor growth and progression, when expressed in cancer-associated fibroblasts [48]. The identification of VEGFA as relevant target for miR-15 in the context of UVB-induced senescence is consistent with these data. On the other hand, mRNA levels of E2F1 and Bcl2 were downregulated in UVB-treated cells (Additional file 4: Table S1 and data not shown), although three miRNAs known to target E2F1 (miR 20a, 20b and 93) and one miRNA known to target Bcl2 (miR-15a) were all significantly downregulated in UVB-treated cells (Figure 3). It is conceivable that, in these cases, additional regulatory processes take place, which remain to be identified. A particular complication in the interpretation of the current results lies in pleiotropic effects of UVB irradiation, which are known to affect the expression of selected miRNAs and mRNAs (also shown here) but also may influence protein translation [49] and/or protein stability [50]. Accordingly, the assignment of miRNAs and gene expression changes reflects plausibility rather than a strict mechanistic dependence. Bioinformatics analysis and preliminary validation analysis performed in the current study highlighted several miRNA targets as potential modulators of UVB-induced fibroblast senescence that were not previously described, including the cdk inhibitor CDKN2B and the transcription factors RUNX1 and RARB. Whereas these findings establish CDKN2B, RUNX1 and RARB as functional target genes for miR-15a and miR-20A, respectively, the binding of these microRNAs to the 3'-UTR of the target genes remains to be confirmed by additional experiments.

### Regulation of UVB-induced senescence: the role of miR-101 and Ezh2

The here reported data demonstrate for the first time the implication of miR-101/Ezh2 signaling in UVB-induced senescence of human dermal fibroblasts. In control experiments, upregulation of miR-101 and the concomitant downregulation of Ezh2 was also observed in two independent HDF strains, HFF-1 and PFF (Additional file 3: Figure S3), indicating that the regulation of these components is a conserved feature in the senescence response of human dermal fibroblasts to UVB irradiation. The inverse relationship between miR-101 and Ezh2 expression levels was noticed before [51,52] and Ezh2 has been shown by others to play a role in cellular senescence [53,54]. When this communication was under revision, it was reported by others that miR-101 controlled Ezh2 function in cellular senescence of mouse embryonic fibroblasts [55]. In the present communication, functional interactions between miR-101 and Ezh2 in UVB-induced senescence of HDF were analyzed in more detail. For example, we tried by knockdown of miR-101 to rescue the expression levels of Ezh2 in UVB irradiated cells. Although knockdown of miR-101 was very efficient also in UVB treated cells, no corresponding increase in the level of Ezh2 mRNA or protein was observed, suggesting that expression of the Ezh2 gene is regulated by additional signals, which remain to be established. It is conceivable that p53 is responsible for the effect, since p53 was shown to suppress the Ezh2 gene promoter [56,57]. Conversely, we also tried to rescue the phenotype of UVB-induced senescence by overexpression of Ezh2 from a lentiviral overexpression vector. Whereas Ezh2 mRNA was significantly upregulated in infected cells, this did not lead to any detectable increase of Ezh2 protein levels, suggesting that overexpression of Ezh2 protein is not well tolerated, at least in HDF. The mechanisms underlying downregulation of Ezh2 in this cell type remain to be understood. After UVB irradiation, Ezh2 protein levels were rapidly reduced, even prior to the induction of miR-101 (Figure 3), strongly suggesting that several different UVB-dependent pathways converge to downregulate Ezh2, the upregulation of miR-101 being just one of several triggers for this process.

## Conclusions

We report here a comprehensive screen for microRNAs and mRNAs regulated during UVB-induced senescence in human diploid fibroblasts. Using advanced bioinformatics solutions, we identified a network of miRNA-mRNA interactions mediating UVB-induced senescence in this cell type, providing a rich resource for future data mining. The data reported in this communication illustrate the regulation of five distinct miRNAs during UVB-induced cellular senescence. Together the results obtained in this study suggest important roles for microRNAs miR-15, miR-20a/b, miR-93 and miR-101, and their mRNA targets, during UVB-induced senescence of human diploid fibroblasts.

## Methods

### Chemicals

All chemicals were purchased from Sigma, unless indicated otherwise.

### Cell culture

Human diploid foreskin fibroblasts (HDF) were either purchased from ATCC (Manassas, VA) (HFF-1 #SCRC-1041; HFF-2 #SCRC-1042) or isolated from newborn foreskin (PFF) in our laboratory, as described [58]. Cells were used at passage 6 (HFF-2, PFF) and passage 10 (HFF-1). HDFs and human osteosarcoma cells (U2-OS; obtained from ATCC, Manassas, VA) were cultured in the same way in DMEM (Sigma) as described [59]. The cumulative population doublings (cPDL) were calculated using the following equation: cPDL = (log(A) – log(B))/0.301 (A: number of cells at the end of one passage; B: number of cells that were seeded at the beginning of one passage).

### SA-β-galactosidase staining

Senescence-associated-β-galactosidase (SA-ß-gal) staining was used to determine the senescent status of the cells. To stain for SA-β-gal, cells were grown on 6-well plates and washed three times with PBS. Afterwards, the cells were fixed with 2% formaldehyde and 0.4% glutaraldehyde in PBS for 5 minutes at room temperature. Cells were washed three times with PBS and prepared for staining as described previously [59]. Therefore, cells were covered with staining solution (150 mM NaCl, 2 mM MgCl, 5 mM potassium ferricyanide, 5 mM potassium ferrocyanide, 40 mM citric acid, 12 mM sodium phosphate, pH 6.0, adding 1 mg/mL 5-bromo-4-chloro-3-indolyl-b-D-galactoside [X-gal] directly before use) and incubated for 24 h at 37°C without light exposition. The reaction was stopped by washing off the staining solution with PBS. Cells were covered with PBS and blue staining indicating the presence of SA-*β-gal* can be detected under the microscope. To calculate the percentage of SA-*β-gal* positive cells, stained cells were counted and related to the total cell number.

### UVB treatment

For UVB treatment fibroblasts were seeded out in 10 cm dishes (Greiner Bio One, Austria) at a density of 6 × 10^5^ (UVB) and 3 × 10^5^ (control). Cells were washed with HBSS (Sigma) and covered with 2 ml HBSS. To calculate the irradiation time, power per area [W/m^2^] was measured by a UVX radiometer (Thermo Fisher) and the following equation was used: irradiation time [s] = energy per area [J/m^2^] divided by power per area [W/m^2^]. The output of a Philips TL20W/01 lamp (Philips, The Netherlands) for 10 cm dishes was determined as 14.2 ± 0.5 W/m^2^. To test the sublethal dosage, cells were irradiated with 3000, 3500, 4000 and 5000 J/m^2^. For experiments, cells were irradiated twice a day with a dose of 4000 J/m^2^ for 4 consecutive days, where irradiation time was 282 ± 10 s. UVB treatment of miRNA knockdown cells were performed in 6-well plates at a density of 1.3 × 10^5^ cells (UVB) and 5 × 10^4^ cells (control). The output of a Philips TL20W/01 lamp for 6-well plates was determined as 17.3 ± 0.5 W/m^2^ and irradiation time was 231 ± 6 s. After the first (day 1) and the last (day 4) irradiation, as well as after day 7, day 9 and day 15 after the first irradiation, cells were lysed and RNA and protein were isolated.

### Protein isolation

For the preparation of whole cell lysates, HDFs were washed twice with cold PBS and scraped off on ice in lysis buffer (50 mM Tris–HCl, 150 mM NaCl, 1% NP-40, 0.25% Na-deoxycholate, 1 mM EDTA, 100 nM Na_3_VO_4_, 1 mM NaF, 10 mM β-glycerophosphate, pH 7.4) from the 6-well plate or 10 cm dish with a rubber policeman. Cells were three times deep-frozen in liquid nitrogen and thawed and further kept on ice for 30 minutes. After centrifugation at 20,000 × *g* for 10 minutes at 4°C, supernatant was used to determine the protein concentration by DC Protein Assay Kit (Biorad, Austria).

### mRNA and miRNA isolation

Total RNA was isolated using either TRIzol® Reagent (Invitrogen; for miRNA) or the RNeasy® Mini Kit (Qiagen; RNA) according the manufacturer’s protocol. For the isolation of RNA with TRIzol® Reagent cells were trysinized, centrifuged, washed with PBS and re-suspended in 1 ml TRIzol® Reagent. After 5 minutes incubation at room temperature 200 μl of Chloroform was added and vigorously vortexed. After 3 minutes incubation at room temperature a centrifugation step was performed for 10 minutes at 14 000 × *g* at 4°C. The aqueous phase was transferred to a micro-centrifuge tube and mixed with 500 μl isopropanol and incubated at room temperature for 10 minutes to precipitate the RNA. After 10 minutes centrifugation at 14 000 × *g* (4°C) the supernatant was discarded and the pellet was washed with ethanol (70%) and centrifuged again for 5 minutes at 6 000 × *g* (4°C). The supernatant was discarded, the pellet air dried and re-suspended in 30 μl of RNAse free water. Using the RNeasy® Mini Kit up to 5 × 10^6^ cells were trypsinized and lysed in 350 μl RLT buffer (including 10 μl β-mercaptoethanol per ml). The lysate was homogenously mixed by pipetting up and down a few times. One volume of 70% ethanol was added, mixed by pipetting and transferred to RNeasy spin column. After a centrifugation step at 12 900 × *g* for 30 seconds at room temperature, three washing steps were performed. First 700 μl RW1 buffer, two times 500 μl RPE (one volume RPE added to four volumes ethanol) whereas the last washing step was carried out for 2 minutes at maximum speed to dry the RNA-binding membrane. RNA was eluted using 30 μl of RNAse-free water. RNA concentration was quantified by photometric measurement at 260 nm and 280 nm.

### Immunoblotting

Equal amounts of protein were subjected to SDS gel electrophoresis (10–12.5% SDS/polyacrylamide gel) and transferred to PVDF membrane by wet electro-blotting (300 mA, 1 h) using the standard Western blot protocol. The membranes were blocked with 5% skim milk in PBS-T (Phosphate buffered saline + 0.1% Tween 20) or in 5% BSA in PBS-T for the detection of phosphorylated proteins for 1 h at room temperature. Primary antibodies were incubated for 1 h at room temperature or overnight at 4°C. After two times of washing with PBS-T, the second antibody was incubated for 45 min at room temperature. After a few washing steps with PBS-T, immune-reactive proteins were detected using an enhanced chemiluminescence system (ECL+, Amersham Life Science, Germany). The following antibodies were used: mouse monoclonal anti-p21^WAF1^ (Pharmingen, #556430) mouse monoclonal anti-pRb (Pharmingen, #554136), mouse monoclonal anti-p53 (Santa Cruz, #sc-126), rabbit polyclonal anti-GAPDH (Santa Cruz, #sc-25778), rabbit polyclonal phospho-p53 (Ser15; Cell Signaling, #9284), mouse monoclonal α-Tubulin (Sigma, #t-5168), mouse monoclonal ant-Cyclin D1 (Neomarkers, #MS-210-P) and mouse monoclonal anti-Ezh2 (BD Biosciences, #612666), mouse monoclonal anti-p16^INK4A^ (BD Biosciences, #511325GR), rabbit polyclonal anti-p15^INK4b^ (Abcam, #ab53034), mouse monoclonal anti-RUNX1/AML1 (Abcam, #ab54869), rabbit monoclonal anti-retinoic acid receptor beta – RARB (abcam, #ab124701). As secondary antibodies, polyclonal antibodies from Dako were used. For positive control, U2-OS cells were transiently transfected with a pcDNA3.1 vector carrying the sequence of either Ezh2 or p16^INK4a^.

### Real-time q-PCR analysis of mRNAs

RNA was isolated using the RNeasy® Mini Kit (Qiagen) and quantified as described above. For cDNA synthesis 0.4-1 μg of total RNA was reverse transcribed with the Transcriptor First Strand cDNA Synthesis Kit purchased from Roche Applied Science and diluted 1:4. Amplification of Ezh2 mRNA was carried out with the following primers: (forward) 5’-CAT TCG GTA AAT CCA AAC TGC-3’ and (reverse) 5’-CGA CAT ACT TCA GGG CAT CA-3’. RUNX1 (Runt-related transcription factor 1) (forward) 5’-CCC TCG TGC CTC CCT GAA CCA-3’ ; (reverse) 5’-GGC TGG GGA GAG GGA TGG ACA-3’. VEGFA (Vascular endothelial growth factor A) (forward) 5’-TTT GCT TGC CAT TCC CCA C-3’; (reverse) 5’-GCT CTT GCT ACC TCT TTC CTC-3’. CDKN2B (Cyclin-dependent kinase 4 inhibitor B) (forward) 5’-GAT GAG GAC AAT GAG GCA AAG-3’ ; (reverse) 5’-TGG GAA GAA AAG CAA GAC AAC-3’. NOTCH3 (Neurogenic locus notch homolog protein 3) (forward) 5’-TCA TCC TCT TCT CTT TCC ACC-3’; (reverse) 5’-TCC CAG ACT CTT CAC AAG AC-3’. As a housekeeper B2M (β-2 microglobulin) was amplified with the following primers: (forward) 5’-GAA TTC ACC CCC ACT GAA AA-3’ and (reverse) 5’-CTC CAT GAT GCT GCT TAC A-3’ and GAPDH (Glyceraldehyde 3-phosphate dehydrogenase) (forward) 5’-GAG TCA ACG GAT TTG GTC GT-3’ ; (reverse) 5’-GAT CTC GCT CCT GGA AGA TG-3’. Real-time q-PCR was performed in duplicates using the SYBR Green I Master Mix in the LightCycler® 480 Instrument (Roche Applied Science).

### Real-time q-PCR analysis of microRNAs

RNA was extracted with TRIzol® Reagent (Invitrogen, as described above). Using the Universal cDNA Synthesis Kit (Exiqon, Denmark) cDNA synthesis was performed according the manufacturer’s protocol. To perform real-time PCR SYBR Green master mix and specific primer for miR-101 (hsa-miR-101, LNA PCR primer set, UniRT; Exiqon, Denmark) were used. Expression levels of miR-101 were normalized to endogenous 5S rRNA (Exiqon, Denmark). In addition, 7 other microRNAs (hsa-miR-155, hsa-miR-15a, hsa-miR-17, hsa-miR-20a, hsa-miR-20b, hsa-miR-34a, hsa-miR-93) were chosen for qPCR confirmation of array data using the Taqman qPCR platform (Life Technologies). In brief, specific reverse transcription (RT) reactions were performed with each microRNA primer and RNU44 as endogenous control using 10 ng of total RNA as input material. Following RT, qPCRs were run in 4 replicates using the Taqman Universal Mastermix (Life Technologies) on a Rotor-Gene Q (Qiagen) according to the manufacturer’s protocol. Data analysis was performed using the ddCt method.

### Genome-wide RNA profiling (microarray)

Total RNA was isolated using the RNeasy® Mini Kit (Qiagen, as described above). After quantification RNA was sent to The Microarray Facility (Tübingen, Germany). The obtained data set was analyzed using CARMAweb 1.5 (https://carmaweb.genome.tugraz.at/carma/). A cut-off of 1.5 fold change was used to determine the total number of up- and downregulated genes.

### Low density array (TaqMan q-PCR)

RT-q-PCR was performed using Taqman® Low Density Array technology (Applied Biosystems). A total of 93 candidate and three housekeeper genes were used to design Taqman® custom array. According to the manufacturer’s protocol q-PCR was performed, as described [60].

### Locked nucleic acid (LNA)-miRNA microarray

For miRNA expression profiling, LNA-miRNA microarrays (Exiqon, Denmark) consisting of 559 human and 170 mouse LNA-modified probes [61] against miRNAs annotated in Sanger miRBase v9.2 [62] as well as 77 not yet annotated probes (miRPlus, Exiqon, Denmark) were used as previously described [16]. In brief, total RNA extracts were end-labeled using Cy3 dye and hybridized against a common reference RNA-pool end-labeled with Cy5 on a Tecan HS 400 hybridization station (Tecan, Austria). Arrays were scanned at 10 μM resolution (Axon Genepix 4000B, Axon Instruments) and raw intensities were extracted using GenePixPro 4.1 software (Axon Instruments). The acquired array signal data was further processed under R/Bioconductor using linear models for microarray analysis [63] and differential expression between control and UVB treated cells was calculated using t-statistics and p-value adjustment to multiple testing according to Benjamini-Hochberg.

### Stable overexpression and knockdown of Ezh2

For the overexpression of Ezh2 the lentiviral pLenti6/V5-DEST Gateway vector (Invitrogen) was used. Cloning included the TOPO cloning of Ezh2 into pENTR/D-TOPO. This vector was used to introduce the Ezh2 coding sequence into pLenti6/V5-DEST by recombination to generate the transfer vector pLenti6-Ezh2 (for further details see Invitrogen’s *ViraPower Lentiviral Expression System* manual). The GFP-control vector was generated in the same way. As a transfer vector for knockdown of Ezh2 lentiviral pLKO.1-TRC short-hairpin vector were purchased from Addgene/Open Biosystems (United Kindom). The following sequences were chosen: pLKO-73 within the 3’UTR of Ezh2 (5’-TAT TGC CTT CTC ACC AGC TGC-3’) and pLKO-75 within the coding region (5’-CCA ACA CAA GTC ATC CCA TTA-3’). As a control the empty vector was used. For packaging of the lentivirus in HEK293FT cells (Invitrogen), 3 μg of the corresponding overexpression or knockdown vector was combined with 7.5 μg vector backbone psPAX2, 2.5 μg envelope encoding plasmid pMD2.G. HEK293FT cells were cultivated in T75 flasks to 90% confluence and transfected with the mixture using Lipofectamine 2000 (Invitrogen). The next day medium was replaced by 10 ml of growth medium without antibiotics. After 48 hours (day 5) the supernatant was harvested, centrifuged at 300 × g for 5 minutes at room temperature and filtered through a 0.45 μm PVDF filter (Millipore, Vienna, Austria). Afterwards, the virus containing supernatant was concentrated using Polyethylene glycol. Therefore, to one volume Polyethylene glycol solution (50 mM Polyethylene glycol, 41 mM NaCl, autoclave, pH =7.2; PEG) four volumes of supernatant was added and incubated for 2 hours at 4°C, carefully mixing every 20–30 minutes by inverting. The solution was centrifuged at 1500 × g for 30 minutes at 4°C and a white pellet should be visible. After aspirating the supernatant an additional centrifugation step was carried out at 1500 × g for 5 minutes at 4°C to collect remaining PEG which is aspirated carefully. The white pellet was re-suspended in medium by pipetting up and down and vigorously vortexing for 20 to 30 seconds. As a guideline 500 μl for one T75 flask was used, aliquoted to 100 μl and stored at −80°C. The titer of the concentrated lentiviral supernatant was determined by seeding 5 × 10^4^ U2-OS in 6-well plates with 8 mg/mL hexadimethrine bromide (polybrene; Sigma-Aldrich) as transduction enhancer. A stock of 4 mg/mL of polybrene was prepared by resolving polybrene in sterile water and filtered through a 0.22 μm sterile filter. The following day the medium was replaced by 2 ml of DMEM. After day 3 the selection was started. For overexpression selection 10 μg/ml Blasticidin, for knockdown 500 ng/ml puromycin was added to each well. The antibiotic containing medium was replaced every second day. Approximately 6–7 days after starting the selection the untransduced cells are dead. For the staining the cells are washed three times with PBS, covered with crystal violet and incubated for 5–10 minutes at room temperature. Wells are washed twice with ddH2O and air dried. The colonies are counted and multiplied with 1 000 and the corresponding dilution. This procedure allows to calculate transfection units (TU) of the virus/ml.

### Overexpression and knockdown of miRNAs

To achieve the overexpression of microRNA in HDFs, cells were reverse transfected with Pre-miR™ miRNA Precursor for miR-15a, miR-20a, miR-93, miR-101, and Pre-miR™ miRNA Precursor Molecules-Negative Control #2 for negative control (Applied Biosystems, Austria) using siPORT™ NeoFX™ Transfection Agent (Ambion, Austria) according the manufacturer’s protocol. Cells were trysinized, counted and 6 × 10^4^ cells were centrifuged to be re-suspended in 1.8 ml medium. The transfection reagent siPORT™ NeoFX™ and Opti-MEM medium (Invitrogen) were adjusted to room temperature. Opti-MEM medium was mixed with Precursors and with the transfection reagent and incubated for 10 minutes incubation at room temperature. Both reagents are mixed and additionally incubated at room temperature for 10 minutes. Afterwards, the mixture is added together with the cell suspension to 6-well plates [22]. Cells were UVB treated as described above. Experiments were done in 6-well plates with 1.3 × 10^5^ (UVB) and 5 × 10^4^. After the last exposure, cells were trypsinized and prepared for transfection. For miR-101 knockdown, cells were reverse transfected with miRCURY LNA™ microRNA Power Inhibitor and miRCURY LNA™ microRNA Power Inhibitor Negative Control A for negative control (Exiqon, Denmark) using siPORT™ NeoFX™ Transfection Agent (Ambion, Austria) according the manufacturer’s protocol. UVB treated cells were trypsinized, counted, centrifuged and re-suspended in 1.8 ml medium. Opti-MEM medium was mixed with the miRNA inhibitor or control and with the transfection reagent and incubated for 10 minutes at room temperature. The protocol was continued as described above. For both, Power Inhibitor and Negative Control A 15 nM (day 4 and day 7) were used.

### Transduction of cells by lentiviral particles

For transduction 5 × 10^4^ human diploid fibroblasts (HDFs) are seeded to 6-well plates the day before (day 1). A multiplicity of infection of two together with 8 μg/ml polybrene as transduction enhancer was used in a total of one milliliter. The medium was changed the next day and selection was started at day 3. For the selection of Ezh2 overexpressing cells 10 μg/ml blasticidin, for Ezh2 knockdown cells 500 ng/ml puromycin was used.

### Bioinformatics analysis for predicting miRNA target genes

For miRNA target prediction the following tools were used: TargetScan [64], PicTar [65], miRanda [66], PITA [67], ElMMo [68], RNA22 [69], DIANA-microT [70] and GenMiR++[71]. The predictions of these tools reflect miRNA:mRNA pairing, site location, conservation, site accessibility, multiple sites and expression profile [72]. The results were visualized using in house developed tools (Genesis [73] and ClueGO [74]) as well as publicly available tools: Cytoscape [75].

### Statistical analysis

We used limma package [63] for miRNA microarray analysis. T-test and adjusted p-value were used to identify genes differentially expressed between control and UVB treated cells. Furthermore, we also investigated the linear relationship between miRNAs and their high confidence target genes by using Pearson correlation coefficients. All analyses were done with the use of statistical software programs R/Bioconductor.

### Pathway analysis and network visualization

The data set obtained from the genome-wide RNA profiling was uploaded to Ingenuity Systems (http://www.ingenuity.com/) and pathway analysis was done. The visualization of the network was done using the open-source Cytoscape software platform [75] for visualizing biomolecular interaction networks. The color and shape of nodes are based on different node attributes available for the analyzed dataset. The red triangles, purple circles and orange diamonds in the network are indicating miRNAs, target genes, and transcription factors, respectively. Edges represent correlation between miRNAs and mRNAs, the color of the edges designate the type of interaction. Red is for positive and green is for negative correlation. The biological functions triggered by the miRNA through its target genes were visualized using ClueGO [74] a Cytoscape plugin.

## Abbreviations

ECL: Enhanced chemoluminescence; Ezh2: Enhancer of zeste homolog 2; HBSS: Hanks’ balanced salt slution; HDF: Human diploid fibroblasts; MAPK: Mitogen-activated protein kinase; miR: microRNA; PBS: Phosphate-buffered saline; pRb: Retinoblastoma protein; PDL: Population doublings; RT-qPCR: Real-time quantitative PCR; SA-ß-gal: Senescence-associated ß-galactosidase; shRNA: Small hairpin RNA; UVB: Ultraviolet light band B.

## Competing interests

The authors declared that they have no competing interests.

## Authors’ contributions

PJD, ZT and JG designed the research. EH performed the transcription factor binding sites analysis. MH, RM and MS did the Locked nucleic acid (LNA)-miRNA microarrays for determination of microRNA levels. UVB irradiation experiments and collection of samples were done with the help of MN by RG. RG, AP, and MC performed microRNA knockdown and overexpression experiments, determination of protein levels and bioinformatics. PC analyzed data using bioinformatics tools. Cloning of the Ezh2 vector and the calculation of expression levels of genes of the genome-wide RNA profiling and low density arrays were done with the help of CM, MN and LM. PJD and RG wrote the manuscript. All the authors read and approved the final manuscript.

## Supplementary Material

Additional file 1: Figure S1 Growth characteristics of UVB treated cells. Human diploid fibroblasts (HDF) were UVB treated in 10 cm dishes with 4000 J/m^2^ as described. **(A)** Cumulative population doubling (cPDL) of UVB treated and untreated control cells were calculated at the indicated time points as described in material and methods. **(B)** To determine the senescence status of HDFs, cells were stained for SA-*β-gal* at day 9 after the first irradiation. Bars indicate the relative percentage of SA-*ß-gal*-positive cells (± SD); results were derived from three independent experiments. **(C)** Protein was isolated from UVB irradiated and control cells at the indicated time points. Defined protein levels were determined by standard Western blot analysis. Experiments were performed in duplicates. As were used, as indicated. As positive control for the right panel, lysates from mock-transfected (U2-OS pX) and p16INK4A transfected (U2-OS p16) U2-OS osteosarcoma cells were used, as indicated. cPDL: cumulative population doublings; Co: control. Click here for file

Additional file 2: Figure S2 Senescence status of HFF1 and PFF. HFF1 and PFF were UVB irradiated in 6-well lates with 3000, 3500, 4000, 4500 and 5000 J/m^2^ as described. **(A)** Cumulative population doublings (cPDL) of UVB treated and untreated cells were calculated at the indicated time points as described in material and methods. **(B)** Cells were stained for SA-*β-gal* at d9 after the first irradiation to determine the senescence status. Bars indicate the relative percentage of *β-gal*-positive cells (± SD); results were derived from three independent experiments. Click here for file

Additional file 3: Figure S3 Protein levels of UVB irradiated HDF. Protein was isolated from UVB irradiated and control cells at the indicated time points. Protein levels were analyzed by standard Western blot. Experiments were performed in triplicates. In the lower panels, controls for the Western blots are provided as follows: *Left panel*: p-p53 Western blot in untreated vs. cisplatin-treated HFF-1. *Middle panel*: Ezh2 Western blot in U-2OS cells transfected with pcDNA3-Ezh2 or empt vector (EV), as indicated. Right panel: p16INK4A Western blot iin U-2OS cells transfected with pcDNA3-p16INK4A or empty vector (EV), as indicated. Click here for file

Additional file 4: Table S1 Genome-wide transcriptome analysis after UVB treatment. RNA was isolated from UVB treated and control cells. Genome-wide RNA profiling (microarray) was performed at different time points. A cutoff of > 1.5 fold regulation was used for the result of day 7 and day 9 after UVB irradiation. * indicates significant changes in gene expression at day 9, derived from three independent Affymetrix chip experiments. Click here for file

Additional file 5: Table S2 microRNA expression levels after UVB treatement. miRNA was isolated from UVB treated and control cells. miRNA expression levels were determined by Locked nucleic acid (LNA)-miRNA microarray. A cut-off of > 1.5 fold regulation was used. Adjusted p-values of ≤ 0.05 are in black colour. Click here for file

Additional file 6: Figure S4 Validation of selected miRNA regulatory interactions. miR-15a and miR-20a were overexpressed in HDF as indicated. Extracts were prepared from cells overexpressing the indicated miRNAs and probed with antibodies to RUNX1, CDKN2B, and RARB, as indicated. Click here for file

Additional file 7: Figure S5 Depletion of Ezh2 induces premature senescence in HDFs. Knockdown of Ezh2 was done with two different constructs in HDFs as described. **(A)** cPDLs were calculated for Ezh2 knockdown cells and scrambled transfected cells. Data represents the mean ± SD of three independent experiments. Representative standard western blot analysis of Ezh2 knockdown. **(B)** SA-*β-gal* activity was determined at day 9 after transfection. Bars represent the mean ± SD of three independent experiments. cPDL: cumulative population doublings; scr: scrambled shRNA; shRNA: small hairpin RNA. Click here for file

Additional file 8: Figure S6 Ezh2 protein and mRNA levels after lentiviral Ezh2 overexpression. HDFs were transfected with Ezh2 or GFP overexpression virus. As an additional control HDF wildtype were used. RNA and protein were isolated as described. For quantification of Ezh2 mRNA levels real-time qPCR was performed (left panel). Protein lysates were subjected to SDS-page and analyzed for Ezh2 protein levels by standard Western Blot. Number in brackets represent densitometric data. Click here for file
