# Supplementary figures and images for: Identification of microRNA-mRNA functional interactions in UVB-induced senescence of human diploid fibroblasts
Source: BMC Genomics. 2013 Apr 4;14:224. doi: 10.1186/1471-2164-14-224 (PMC4008267; doi:10.1186/1471-2164-14-224)

A

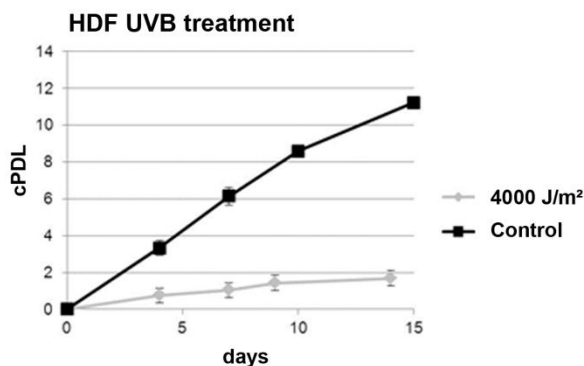

B

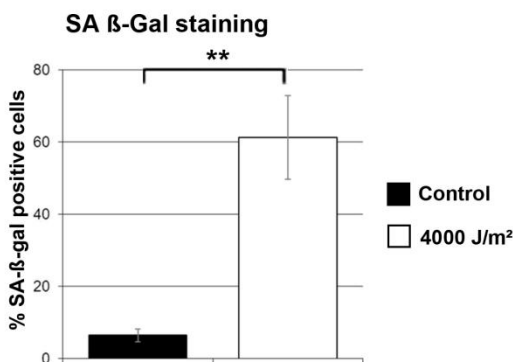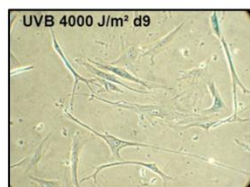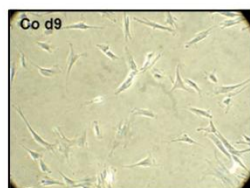

C

### Protein expression in UVB induced senescence

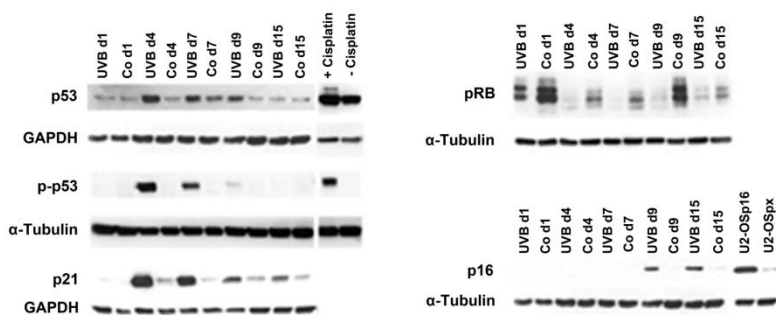

Supplement: Additional file 1: Figure S1 — Growth characteristics of UVB treated cells. Human diploid fibroblasts (HDF) were UVB treated in 10 cm dishes with 4000 J/m2 as described. (A) Cumulative population doubling (cPDL) of UVB treated and untreated control cells were calculated at the indicated time points as described in material and methods. (B) To determine the senescence status of HDFs, cells were stained for SA-β-gal at day 9 after the first irradiation. Bars indicate the relative percentage of SA-ß-gal-positive cells (± SD); results were derived from three independent experiments. (C) Protein was isolated from UVB irradiated and control cells at the indicated time points. Defined protein levels were determined by standard Western blot analysis. Experiments were performed in duplicates. As were used, as indicated. As positive control for the right panel, lysates from mock-transfected (U2-OS pX) and p16INK4A transfected (U2-OS p16) U2-OS osteosarcoma cells were used, as indicated. cPDL: cumulative population doublings; Co: control. [file 1471-2164-14-224-S1.pdf]

**A** HFF1 UVB treatment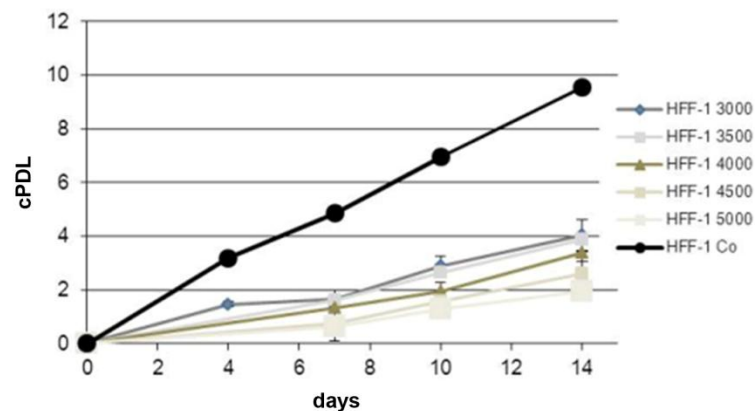**PFF UVB treatment**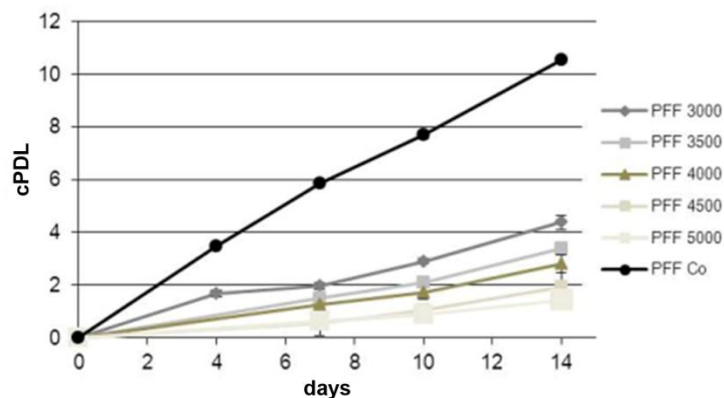**B** SA- $\beta$ -galactosidase (HFF-1)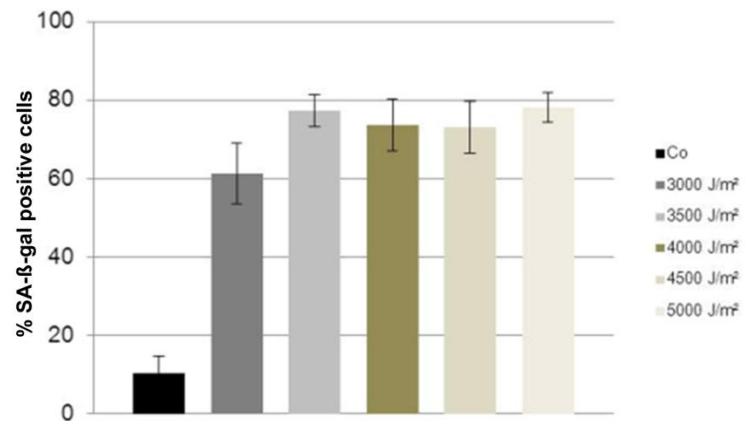**SA- $\beta$ -galactosidase (PFF)**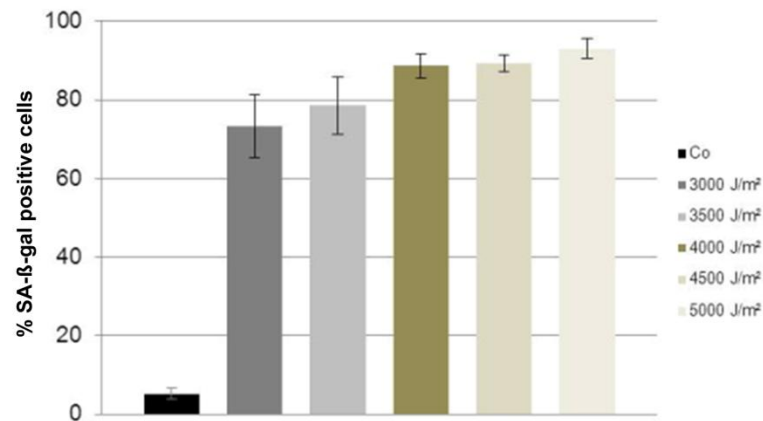

Supplement: Additional file 2: Figure S2 — Senescence status of HFF1 and PFF. HFF1 and PFF were UVB irradiated in 6-well lates with 3000, 3500, 4000, 4500 and 5000 J/m2 as described. (A) Cumulative population doublings (cPDL) of UVB treated and untreated cells were calculated at the indicated time points as described in material and methods. (B) Cells were stained for SA-β-gal at d9 after the first irradiation to determine the senescence status. Bars indicate the relative percentage of β-gal-positive cells (± SD); results were derived from three independent experiments. [file 1471-2164-14-224-S2.pdf]

# Protein expression in UVB induced senescence

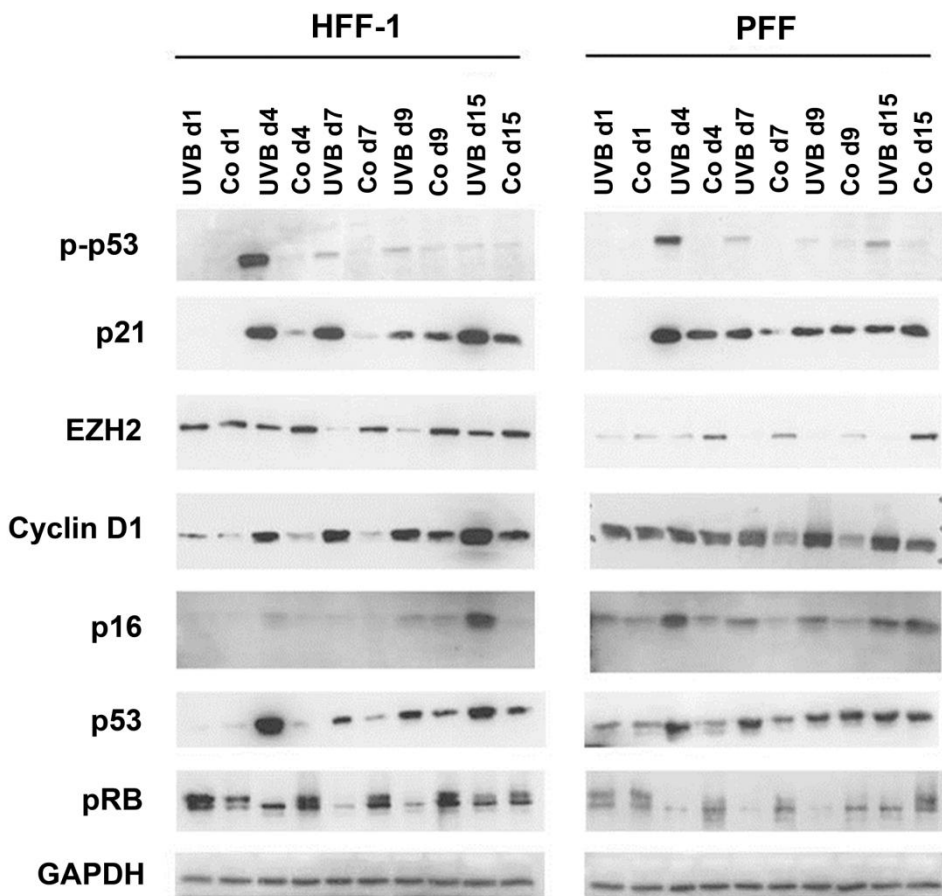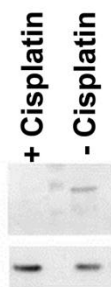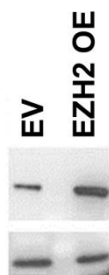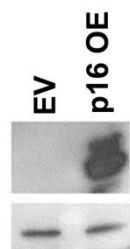

Supplement: Additional file 3: Figure S3 — Protein levels of UVB irradiated HDF. Protein was isolated from UVB irradiated and control cells at the indicated time points. Protein levels were analyzed by standard Western blot. Experiments were performed in triplicates. In the lower panels, controls for the Western blots are provided as follows: Left panel: p-p53 Western blot in untreated vs. cisplatin-treated HFF-1. Middle panel: Ezh2 Western blot in U-2OS cells transfected with pcDNA3-Ezh2 or empt vector (EV), as indicated. Right panel: p16INK4A Western blot iin U-2OS cells transfected with pcDNA3-p16INK4A or empty vector (EV), as indicated. [file 1471-2164-14-224-S3.pdf]

# RUNX1, p15 and RARB protein levels after miR-15a ; miR-20a OE

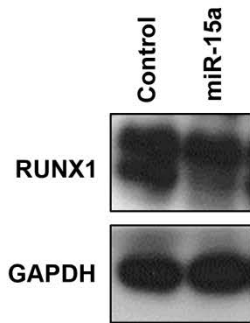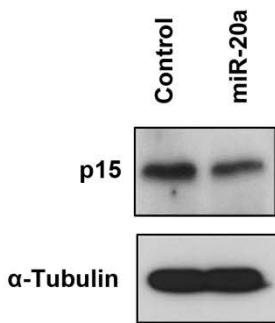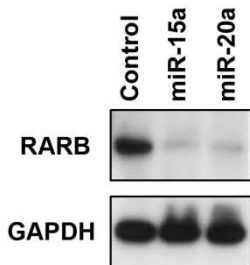

Supplement: Additional file 6: Figure S4 — Validation of selected miRNA regulatory interactions. miR-15a and miR-20a were overexpressed in HDF as indicated. Extracts were prepared from cells overexpressing the indicated miRNAs and probed with antibodies to RUNX1, CDKN2B, and RARB, as indicated. [file 1471-2164-14-224-S6.pdf]

## EZH2 expression levels after OE (virus)

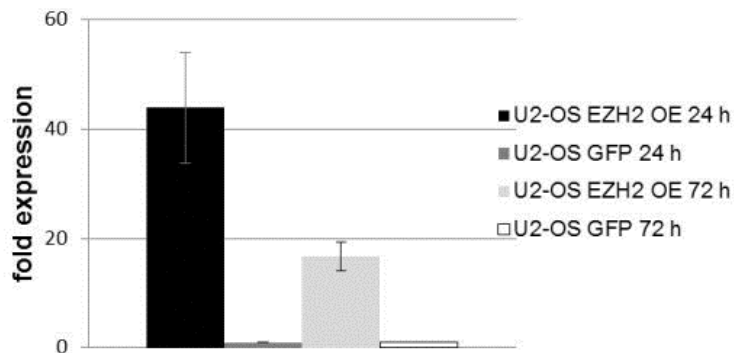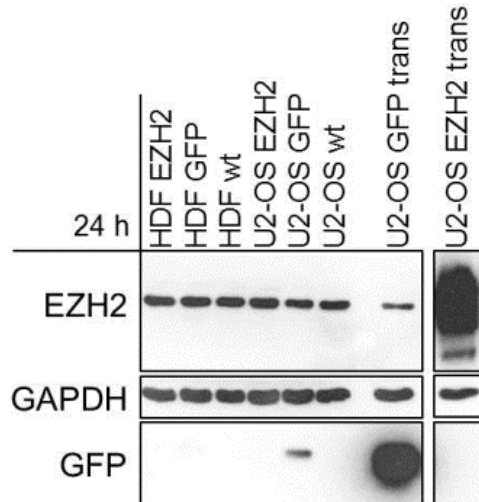

## EZH2 expression levels after OE (virus)

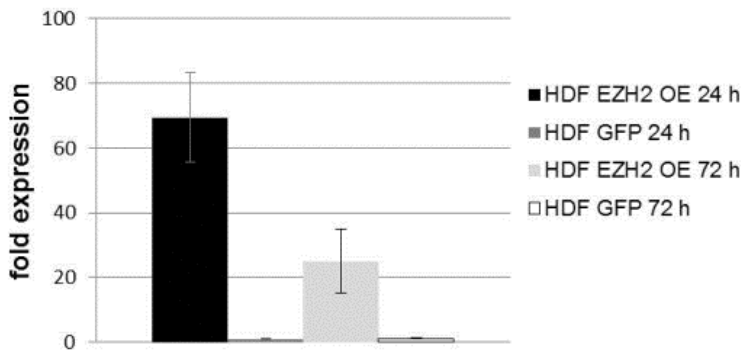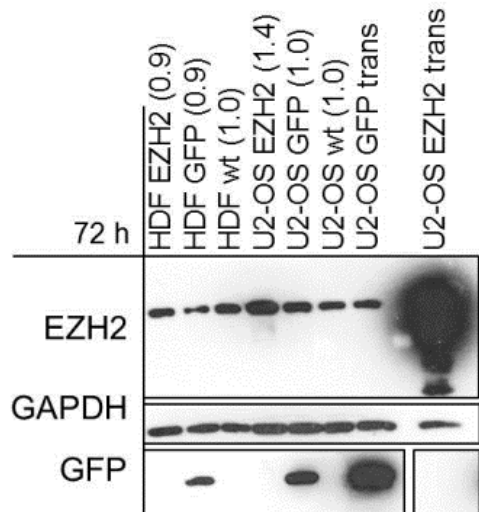

Supplement: Additional file 8: Figure S6 — Ezh2 protein and mRNA levels after lentiviral Ezh2 overexpression. HDFs were transfected with Ezh2 or GFP overexpression virus. As an additional control HDF wildtype were used. RNA and protein were isolated as described. For quantification of Ezh2 mRNA levels real-time qPCR was performed (left panel). Protein lysates were subjected to SDS-page and analyzed for Ezh2 protein levels by standard Western Blot. Number in brackets represent densitometric data. [file 1471-2164-14-224-S8.pdf]
