# Supplementary material for: Identification of microRNA-mRNA functional interactions in UVB-induced senescence of human diploid fibroblasts
Source: BMC Genomics. 2013 Apr 4;14:224. doi: 10.1186/1471-2164-14-224 (PMC4008267; doi:10.1186/1471-2164-14-224)

A

Growth curve: EZH2 KD

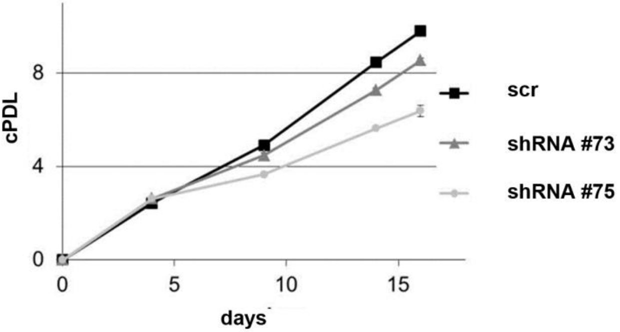

EZH2 protein levels

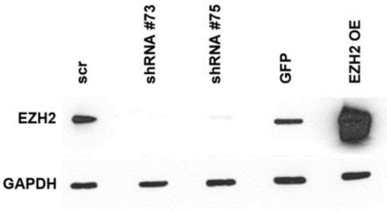

B

Senescence induction by EZH2 depletion

SA ß-Gal staining: EZH2 KD

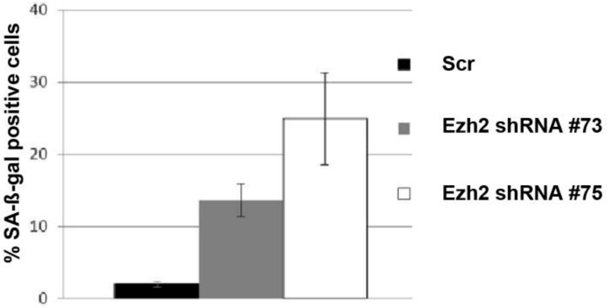

scr | Ezh2 shRNA #73 | Ezh2 shRNA #75

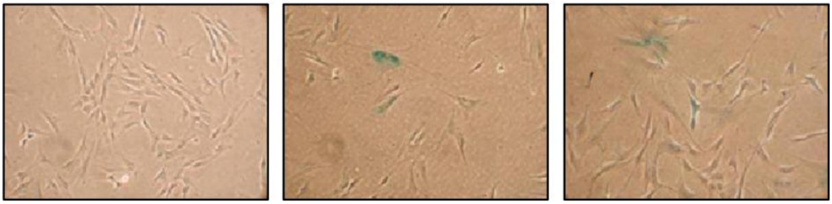

Supplement: Additional file 7: Figure S5 — Depletion of Ezh2 induces premature senescence in HDFs. Knockdown of Ezh2 was done with two different constructs in HDFs as described. (A) cPDLs were calculated for Ezh2 knockdown cells and scrambled transfected cells. Data represents the mean ± SD of three independent experiments. Representative standard western blot analysis of Ezh2 knockdown. (B) SA-β-gal activity was determined at day 9 after transfection. Bars represent the mean ± SD of three independent experiments. cPDL: cumulative population doublings; scr: scrambled shRNA; shRNA: small hairpin RNA. [file 1471-2164-14-224-S7.pdf]
